# Laboratory Assessment of Wastewater as Surveillance Tool for West Nile Virus, United States

**DOI:** 10.3201/eid3213.260616

**Published:** 2026-08

**Authors:** Jessica Spring, Audrey Long, Emily Davis, Shannon R. Matzinger, Allison Wheeler, Shelby L. Lyons, Daniel L. Jacobs, Rory Welsh, Jeffrey W. Mercante, Aaron C. Brault, Holly R. Hughes

**Affiliations:** Centers for Disease Control and Prevention, Fort Collins, Colorado, USA (J. Spring, A. Long, E. Davis, S.L. Lyons, D.L. Jacobs, A.C. Brault, H.R. Hughes); Colorado Department of Public Health and Environment, Denver, Colorado, USA (S.R. Matzinger, A. Wheeler); Centers for Disease Control and Prevention, Atlanta, Georgia, USA (R. Welsh, J.W. Mercante)

**Keywords:** West Nile virus, wastewater, surveillance, viruses, arbovirus, mosquito-borne, vector-borne infections, meningitis/encephalitis, United States

## Abstract

West Nile virus (WNV) is the most common cause of domestic human arboviral infection in the contiguous United States. WNV surveillance across the United States is not comprehensive and is limited. Nonclinical WNV surveillance is variable, and WNV surveillance in humans is limited by the percentage of symptomatic human infections and time it takes to seek healthcare and diagnosis. Wastewater surveillance has been used to detect viruses shed from asymptomatic and symptomatic persons; therefore, we assessed wastewater as a surveillance tool for WNV tracking. We tested archived RNA and raw wastewater from 6 states with documented human illness during the 2023 WNV transmission season for WNV RNA. WNV RNA was detected in 18% (n = 29/158) of samples from Arizona, California, Colorado, Illinois, Indiana, and Nebraska. Our results highlight the ability to detect WNV in wastewater and the potential of wastewater surveillance to augment current surveillance data.

West Nile virus (WNV) is an enveloped, positive-sense RNA orthoflavivirus transmitted by mosquitoes (*1*). Although several lineages of WNV exist, the most common and virulent for humans are lineage I, found worldwide, and lineage II, found in sub-Saharan Africa and Europe (*2*). Most WNV infections are asymptomatic or mild; however, each year ≈1,300 neuroinvasive disease cases are reported in the United States (*3*–*6*). Symptomatic WNV infection is marked by nonspecific symptoms such as headache, body aches, joint pain, vomiting, diarrhea, or rash (*1*,*2*). A severe neuroinvasive disease develops in <1% of those infected with WNV (*1*,*2*). Because no vaccines or therapeutics for WNV have been approved, personal protective behaviors, vector control, and blood and organ donor screening for WNV are essential tools to reduce WNV disease–related illness and death (*7*).

Various modes of surveillance are used to monitor and control WNV transmission, including mosquito, dead bird, and sentinel animal surveillance. However, those surveillance methods are inconsistently used across the United States and rarely performed year-round. Surveillance for WNV in humans includes testing symptomatic persons and monitoring blood donations (*1*,*2*,*8*,*9*). Nevertheless, those methods miss mild infections in persons not seeking medical attention or donating blood, likely underrepresenting the number of WNV cases.

Wastewater surveillance (WS) was successfully implemented to monitor the prevalence and transmission of various viral pathogens including poliovirus, SARS-CoV-2, and monkey pox virus in humans regardless of reported clinical symptoms (*10*–*12*) and sometimes before reported human cases (*13*). WS also proved informative during the COVID-19 pandemic. SARS-CoV-2 virions and viral RNA are shed in patient feces and recoverable in wastewater, which enabled the adaptation of wastewater monitoring to assist in estimating the prevalence of COVID-19 cases and detecting concerning variants (*14*–*16*). Since the COVID-19 pandemic began, WS has rapidly expanded as an effective method to monitor transmission of enteric and respiratory pathogens on a large scale, resulting in multiple countries establishing regional or national level systems (*17*).

Studies have identified measurable levels of WNV RNA in urine from both symptomatic and asymptomatic persons after WNV infection (*9*,*18*–*20*). Because WS was an effective tool for other viruses and WNV has been shown to be shed in urine, we sought to determine the feasibility of WS for WNV. We assessed WNV RNA recovery from spiked wastewater samples and WNV RNA detection from wastewater samples collected in counties with human or nonhuman WNV activity during the 2023 transmission season.

## Materials and Methods

### Virus Growth

We infected confluent Vero cells (American Type Culture Collection, https://www.atcc.org) in 150-cm^2^ flasks with WNV lineage I isolate RO97–50 at a multiplicity of infection of 0.01. We maintained infected cells in yeast extract/lactalbumin media supplemented with 3% (vol/vol) sodium bicarbonate (Invitrogen, https://www.invitrogen.com), 2% (vol/vol) fetal bovine serum (Cytiva, https://www.cytivalifesciences.com), 0.1% (vol/vol) Gentamicin (MP Biomedicals, https://www.mpbio.com), 0.4% (vol/vol) Fungizone (Cytiva), and 1% (vol/vol) Pen/Strep (Invitrogen). We collected the supernatant from infected cells at 80% cytopathic effect and froze the supernatant at −20°C.

### WNV Stability in Contrived Wastewater Samples

We spiked wastewater from the Colorado Department of Public Health and Environment (CDPHE) collected outside of typical arbovirus transmission period (collected March 2023) with 10^4^–10^7^ RNA copies/140 µL of WNV. We included unmanipulated wastewater as a negative control. We pasteurized samples for 30 minutes at 56°C. For time course studies, we left samples at room temperature before pasteurization. We chose a time course to encompass the early time points at which wastewater would be collected and to enable further determination of WNV stability in wastewater. We extracted RNA from 140µL of contrived wastewater samples by using the QIAamp Viral RNA Mini Kit (QIAGEN, https://www.qiagen.com) and eluted in 100 µL of AVE buffer.

### RNA Samples Derived from Wastewater

CDPHE provided 48 deidentified, archived RNA samples derived from wastewater on the basis of a request for available samples collected from regions with documented human WNV cases during the summer of 2023. Wastewater was collected from 2 Colorado counties during the summer of 2023. Nanotrap enhancement reagents (CERES Nano, https://www.ceresnano.com) were used to concentrate 40mL of wastewater following manufacturer’s instructions. RNA had been previously extracted from concentrated wastewater by using the MagMAX Wastewater Ultra Nucleic Acid Isolation Kit (Thermo Fisher Scientific, https://www.thermofisher.com).

### Wastewater Samples and Concentration

The Centers for Disease Control and Prevention’s National WS System biorepository provided 110 archived raw wastewater samples collected during the 2023 WNV transmission season. The wastewater was provided on the basis of a request for available samples that had been collected in counties or states with documented human WNV cases during the 2023 transmission season. 

Volumes of 15 mL per sample were shipped to the Centers for Disease Control and Prevention, National Center for Emerging and Zoonotic Infectious Diseases, Division of Vector-Borne Diseases, Arboviral Diseases Branch (Fort Collins, CO, USA) and stored at −80°C until processing. We concentrated raw wastewater by using polyethylene glycol precipitation as previously described (*21*). We thawed raw wastewater and waited for debris to settle. From the clarified top section of the sample, we extracted 5 mL and heat treated the sample for 30 minutes at 56°C. We mixed the pasteurized wastewater with polyethylene glycol (0.14 g/mL) and NaCl (0.0117 g/mL). We shook the samples at 200 rpm in a MaxQ 4000 Orbital Shaker (ThermoFisher Scientific) for 4 hours at 4°C and then centrifuged the samples at 6,500 × *g* for 30 minutes at 4°C. We discarded the supernatant and resuspended the pellets in 500 µL sterile phosphate buffered saline. We stored the resuspended pellets at −80°C until RNA extraction. We isolated RNA from resuspended pellets by using the QIAamp Viral RNA Mini Kit (QIAGEN) according to the manufacturer’s instructions. We eluted RNA in a total volume of 30 µL AVE buffer (QIAGEN) twice and stored at −80°C.

### Digital Reverse Transcription PCR

We conducted digital reverse transcription PCR (dRT-PCR) to quantify WNV RNA copies by using a QIAcuity One Digital PCR System (QIAGEN) and the QIAcuity OneStep Advanced Probe Kit (QIAGEN) according to the manufacturer’s instructions. We quantified WNV RNA copies by using envelope-specific primers and probe (forward, 5′-TCAGCGATCTCTCCACCAAAG-3′; reverse, 5′-GGGTCAGCACGTTTGTCATTG-3′; probe, 5′-TGCCCGACCATGGGAGAAGCTC-3′) (*22*). For contrived wastewater samples, we loaded 1 µL RNA in a total mixture volume of 12 µL onto an 8.5K Nanoplate (QIAGEN). For RNA provided by CDPHE and RNA extracted from archived wastewater samples, we loaded 10 µL RNA in a total volume of 40 µL onto a 26K Nanoplate (QIAGEN). We included primers and probes specific for pepper mild mottle virus (PMMoV) (PMMoV-FP1-rev 5′-GAGTGGTTTGACCTTAACGTTTGA-3′; PMMoV-RP1 5′-TTGTCGGTTGCAATGCAAGT-3′; PMMoV-Probe1 5′-/56-FAM/CCTACCGAA/ZENGCAAATG/3IABkFQ/-3′) when testing RNA extracted from wastewater as positive extraction control. For the PMMV assay, we loaded 1 µL RNA in a total volume of 12 µL onto an 8.5K Nanoplate (QIAGEN). We included a positive WNV control, a negative wastewater control, and a water control on each plate. We considered samples with >3 positive partitions positive. Negative and water control wells all had <3 positive partitions.

### Real-time Reverse Transcription PCR

We subsequently analyzed samples with >3 positive partitions from dRT-PCR testing by using real-time reverse transcription PCR (RT-PCR). We used the Quantitect probe RT-PCR kit (QIAGEN) to detect viral RNA by using the WNV envelope primers and probe (*22*) according to the manufacturer’s instructions. We used 10 µL of RNA in a total reaction volume of 25 µL. We included positive and negative controls on each plate. We deemed a cycle threshold value <38 positive (*23*). We only considered samples that were positive by both dRT-PCR and real-time RT-PCR methods positive for WNV RNA.

### Surveillance Data

We obtained surveillance data for 2023 from ArboNET, the national surveillance system for arboviral diseases (https://www.cdc.gov/vector-borne-diseases/php/arbonet). State and territorial health departments voluntarily report nonhuman (i.e., positive mosquitoes, sentinel animals, or animal disease cases) and human (disease cases and presumptive viremic donors) data to ArboNET by using standard surveillance case definitions. We compared the timing of positive surveillance events with wastewater testing results by using timelines beginning with the first WNV surveillance event (day 0). Human cases are demarcated by onset date.

## Results

### WNV Stability in Contrived Wastewater

**Figure 1:**
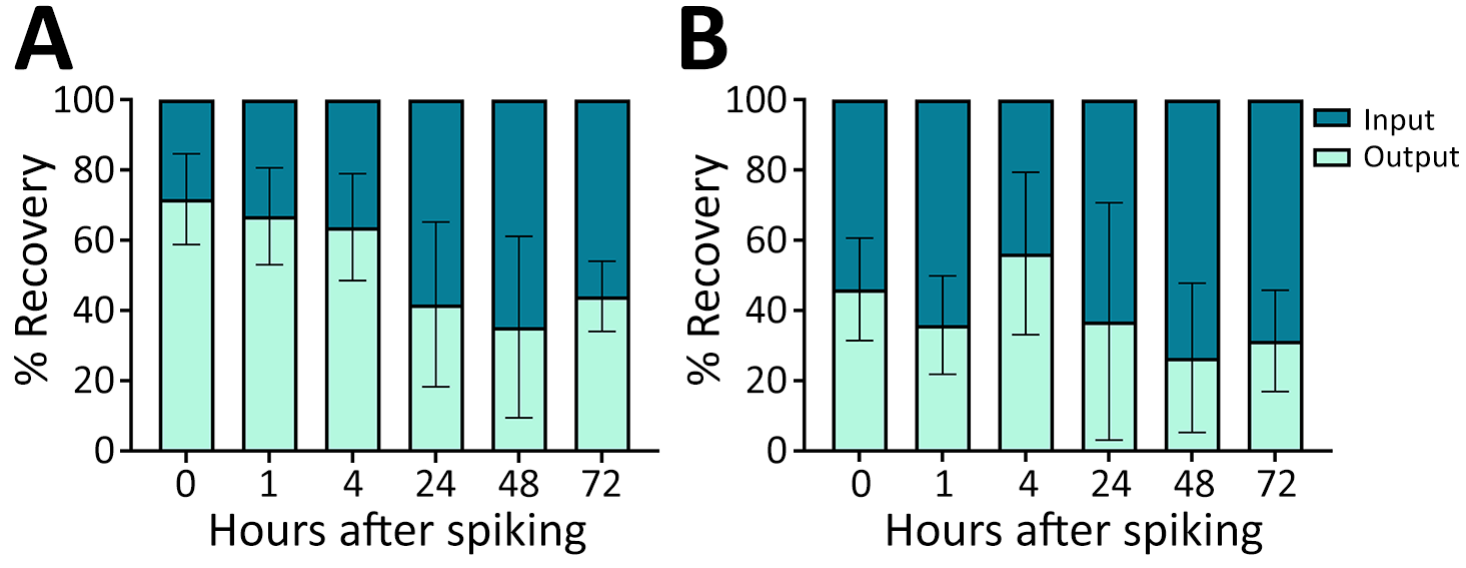
West Nile virus (WNV) RNA detection by digital reverse transcription PCR in spiked wastewater samples up to 72 hours from laboratory assessment of wastewater as surveillance tool for WNV, United States. WNV RNA recovery over time from wastewater spiked with a high concentration of WNV (known yield 10^6^–10^7^ RNA copies) (A) or a low concentration of WNV (known yield 10^4^–10^5^ RNA copies) (B). Data are represented as averages from multiple experiments; error bars represent SD.

We determined recovery of WNV RNA from wastewater via dRT-PCR, which has enhanced resistance to inhibitors in complex matrices like wastewater (*24*). Immediate (0 h) isolation of RNA from wastewater spiked with a high concentration (yields 10^6^–10^7^ RNA copies/140 µL) of WNV resulted in an average of 75% recovery (Figure 1, panel A). Detection dropped to 60% by 4 hours after spiking and 50% by 72 hours after spiking (Figure 1, panel A). As expected, detection of recovered WNV RNA in wastewater spiked with a low concentration (yields 10^4^–10^5^ RNA copies/140 µL) of WNV was lower, with an average of 50% recovery at 0–4 hours, and down to 35% recovery by 72 hours after spiking (Figure 1, panel B). We detected WNV spiked in wastewater down to a limit of 14 copies/µL of input RNA and observed large deviations in recovery particularly at later timepoints, suggesting variability in the degradation of WNV virions and RNA. Nevertheless, those data indicate WNV can be detected in contrived wastewater samples for up to 72 hours after spiking.

### Detection of WNV in Archived RNA

We next investigated whether WNV RNA could be detected in archived RNA derived from wastewater collected during the Colorado arbovirus transmission season of 2023. We tested 48 residual RNA samples from 2 counties for the presence of WNV RNA by dRT-PCR. Five (10.4%) of 48 samples were positive for WNV RNA (Table 1). Of the negative samples, 2 had 2 positive partitions, 9 had 1 positive partition, and 32 had 0 positive partitions. Those data demonstrate WNV RNA can be detected in wastewater by using dRT-PCR.

**Table 1:** Archived RNA samples positive by PCR for West Nile virus RNA in a laboratory assessment of wastewater as surveillance tool, United States*

CDPHE sample	No. positive partitions
1	6
2	6
3	12
4	3
5	4

*CDPHE, Colorado Department of Public Health and Environment.

### Detection of WNV in Archived Wastewater Samples

**Figure 2:**
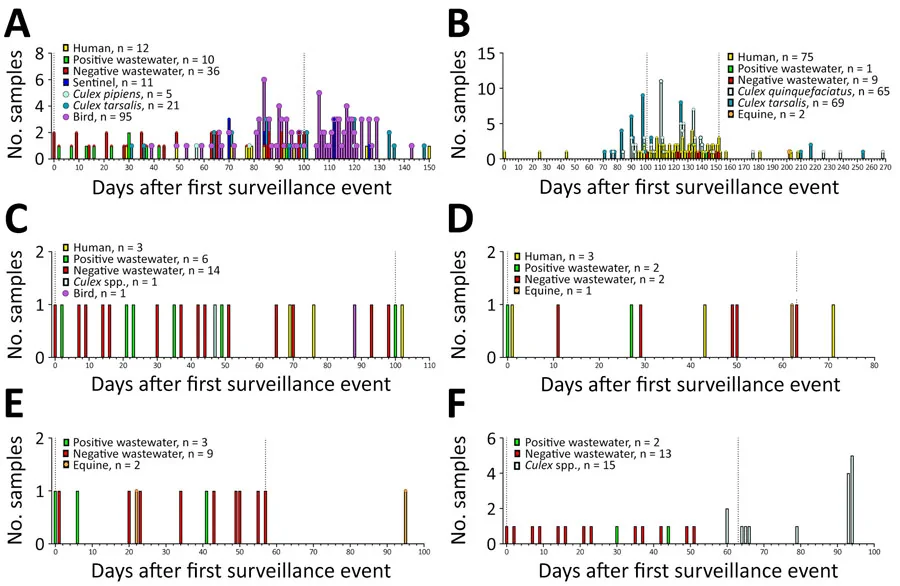
West Nile virus (WNV) RNA detected in wastewater collections from sampled jurisdictions compared with other surveillance methods from laboratory assessment of wastewater as surveillance tool for WNV, United States. A) County A, California, USA. B) County B, Arizona, USA. C) County C, Illinois, USA. D) County D, Nebraska, USA. E) County E, Colorado, USA. F) County F, Indiana, USA. Wastewater samples from each county were tested by digital reverse transcription PCR and real-time reverse transcription PCR for WNV RNA. Wastewater data are compared with the number of other WNV-positive surveillance events reported to ArboNET (y-axis): human, equine, *Culex* mosquito, sentinel, and bird samples. Timeline for samples is dictated as days after first surveillance event (x-axis). Dotted vertical lines mark the time frame for available archived wastewater.

We next examined whether archived wastewater samples collected by the National Wastewater Surveillance System jurisdictions were positive for WNV. We tested wastewater collected during the 2023 WNV transmission season from deidentified counties with or without documented human cases of WNV for the presence of WNV RNA. We then compared results with other reported surveillance data from the same county to determine whether WS could bolster existing surveillance methods (Figure 2).

During the 2023 WNV transmission season, county A in California reported 12 human WNV infections. We tested 46 wastewater samples from the 2 watersheds (data are combined) servicing that county (Table 2). Of those samples, 10 were positive for WNV by dRT-PCR and real-time RT-PCR (Table 3; Figure 2, panel B). Of note, wastewater samples were positive for WNV before positive bird detections by 37 days, sentinel detections by 68 days, and mosquito detections by 29 days (Figure 2, panel A). Furthermore, 5 wastewater samples were positive for WNV up to 27 days before the first documented human WNV case.

**Table 2:** Wastewater sample site details including jurisdiction, population size, and total samples collected in a laboratory assessment of wastewater as surveillance tool for West Nile virus, United States

Jurisdiction	Sewershed population	No. samples
County A, CA	709,694	46
County B, AZ	2,400,000	10
County C, IL	240,000	20
County D, NE	4,800	7
County E, CO	7,300	12
County F, IN	28,000	15

.

**Table 3:** Results for NWSS jurisdiction wastewater samples that were positive by dRT-PCR and real-time RT-PCR collected in a laboratory assessment of wastewater as surveillance tool for West Nile virus, United States*

Sample jurisdiction	First reported nonwastewater surveillance event, d	First reported human disease case, d	RNA copies/5 mL wastewater†	Ct† (*23*)
County A, CA	−27	−27	72.96	36.54
	−22	−22	19.08	32.24
	−16	−16	24.96	35.7
	−13	−13	18.96	37.5
	−6	−6	42.84	35.31
	1	1	184.08	32.44
	1	1	135.72	36.08
	8	8	19.2	35.99
	13	13	30.48	34.97
	64	64	18.96	36.91
County B, AZ	143	143	24.24	36.29
County C, IL	−67	−67	249.72	32.16
	−48	−48	84.84	34.86
	−46	−46	84.84	34.59
	−34	−34	1380	26.91
	−20	−20	38.4	36.53
	31	31	31.68	31.88
County D, NE	−1	−1	44.88	34.14
	26	26	18.24	36.9
County E, CO	−22	NA	28.56	37.09
	−16	NA	57	33.19
	19	NA	18.36	35.26
County F, IN	−30	NA	225.96	32.81
	−16	NA	155.76	34.91

*Ct, cycle threshold; dRT-PCR, digital reverse transcription PCR; NA, not applicable; NWSS, National Wastewater Surveillance System; RT-PCR, reverse transcription PCR. †RNA copies/5 mL of wastewater from dRT-PCR and Ct values from real-time RT-PCR.

County B in Arizona reported 72 human WNV disease cases and 5 asymptomatic presumptive viremic blood donors during the 2023 transmission season. We excluded 2 human cases that did not have reported onset dates from the graph. More than 130 WNV-positive mosquito pools and 2 equine cases were also identified during that time. We assayed 10 wastewater samples for WNV RNA. Although only 1 sample was positive for WNV RNA, it was collected during the time frame when human WNV cases were reported (Table 3; Figure 2, panel B3). The single positive wastewater sample was collected 143 days after the first reported human case, 72 days after the first reported positive mosquito pool, and 59 days before the 2 reported WNV equine cases. The limited number of samples available for testing might have prevented detection of WNV earlier in the season (Figure 2, panel B).

County C in Illinois reported 3 human WNV infections during the 2023 transmission season. Of the 20 wastewater samples available, 6 were positive for WNV (Table 3; Figure 2, panel C). Five wastewater samples were positive for WNV up to 67 days before the first human case and up to 86 days before a WNV positive bird was detected. In addition, 4 wastewater samples were positive for WNV up to 45 days before a single positive mosquito pool.

We tested 7 wastewater samples from county D in Nebraska, which reported 3 human cases of WNV. Of those samples, 2 were positive for WNV RNA (Table 3; Figure 2, panel D), and wastewater was positive for WNV 1 day before the first documented human WNV case and 62 days before a single equine WNV case.

County E in Colorado had no reported human WNV cases during the 2023 transmission season but did report 2 equine cases (Figure 2, panel E). Of 12 wastewater samples available from the county, 3 were positive for WNV RNA, the first of those positive 22 days before the first equine case (Table 3; Figure 2, panel E). County F in Indiana also reported no human cases of WNV during the summer of 2023. However, of the 15 wastewater samples available, 2 samples tested positive for WNV RNA 30 and 16 days before detection of WNV in mosquito pools (Table 3; Figure 2, panel F).

## Discussion

WS has proven a useful tool for monitoring circulation of certain infectious agents, prevalence of bacteria with antimicrobial resistance, and traces of narcotics at a population scale (*25*–*27*). For pathogens transmitted by the fecal–oral route, WS has shown to be valuable for detecting outbreaks, often before widespread transmission and clinical manifestations, leading to swift public health responses (*28*–*30*). 

WNV RNA was detected in both contrived and archived wastewater samples tested in this study. In total, WNV RNA was detected in 18% (29/158) of samples tested. Five archived RNA samples from Colorado were positive for WNV RNA by dRT-PCR. Furthermore, 24 of 110 raw wastewater samples were found positive for WNV RNA by dRT-PCR and real-time RT-PCR. Of note, many positive wastewater samples were collected before documented human infections (Figure 2, panels A, C, D), indicating WS might provide knowledge of WNV circulation before other surveillance methods or in areas with limited WNV surveillance. Early detection of WNV by using WS might be particularly helpful because lag times often occur between WNV activity detected by existing surveillance efforts (if detected at all) and identification of human cases. Counties without vector surveillance might also lack vector control methods. Regardless, information yielded from WS could be used to inform healthcare providers and the public of the risk for WNV transmission. WNV was also detected in wastewater from counties with robust WNV surveillance before reported detection in the environment (e.g., California and Illinois). It is unknown if environmental surveillance was conducted during those times; nevertheless, WS could bolster existing surveillance strategies. Finally, WNV positive wastewater samples were detected in absence of reported human illness (Figure 2, panels E and F). That finding indicates that WS might detect WNV shed from subclinical human infections. Collectively, our data demonstrate wastewater can provide notice of circulating WNV, perhaps even in advance of other surveillance methods. Nevertheless, additional prospective work is needed to determine site specific applicability of WNV WS to augment WNV prevention and control.

Our results, along with those of other studies, support the feasibility of identifying WNV in wastewater. One study found 16 of 21 wastewater samples positive for WNV from counties in Oklahoma (K.G. Kuhn et al., unpub. data, https://dx.doi.org/10.2139/ssrn.4805820). In that study, the samples were deemed positive if 1 droplet was positive, rather than the 3 positive partitions used with dRT-PCR in this article. Compared with another study, our results indicate the liquid fraction of wastewater is likely more sensitive for detection of WNV because only 2% of 601 settled solid samples were positive for WNV RNA (*31*) compared with 10%–30% positivity found among our samples (Arizona, 10%; California, 22%; Colorado, 25%; Illinois, 30%; Indiana, 13%; and Nebraska, 29%).

Other arboviruses, including Zika virus (ZIKV), Japanese encephalitis virus, and dengue virus (DENV), have been successfully detected in wastewater (*32*–*34*). Those wastewater detections occurred around a small cluster of ZIKV cases (*32*), during an acute outbreak of Japanese encephalitis virus (*33*), and in an area with documented cases and emerging risk for DENV (*34*). In contrast, similar studies were not successful in detecting ZIKV during an outbreak or DENV in an endemic region (*35*,*36*). Discrepancies in detecting arboviral RNA in wastewater were potentially caused by a variety of factors including differences among the targeted viruses, the wastewater systems, sample storage and preparation, and assay specificity and sensitivity.

The first limitation of this study is that wastewater is a complex matrix that greatly hindered our attempts to sequence wastewater samples and further confirm samples. Because of wastewater resistance to inhibitors, we relied primarily on dRT-PCR for detection of WNV RNA in wastewater samples. Further work would be necessary to optimize wastewater sequencing for WNV and reduce potential false positive and to standardize detection of WNV in wastewater, which is influenced by collection volumes, instrument use, and defined limits of detection. Second, because archived wastewater samples were not collected specifically to detect WNV transmission, wastewater samples collected during peak WNV transmission were limited and underwent a freeze–thaw cycle that might have degraded WNV RNA. Third, because of sample volume limitations, we extracted RNA from only 5 mL of wastewater, whereas routine surveillance methods use volumes up to 50 mL. The smaller volume might have been inadequate to detect WNV RNA in some instances. Fourth, the detection of WNV RNA in wastewater was compared with WNV detection by other surveillance methods reported to ArboNET. Because data are voluntarily reported to ArboNET, not all instances of WNV detection by other surveillance methods might be included in our datasets. Finally, we lacked information on the type of wastewater system in each jurisdiction. Wastewater systems are generally separate sanitary sewers (collect wastewater only) or combined sewers (collect both wastewater sewage and stormwater runoff). Certain mosquito species are known to breed in storm sewer pipes (*37*). Therefore, we cannot rule out nonhuman sources of WNV RNA in wastewater samples. Knowledge of the type of wastewater system at each collection site might have provided more information regarding the source of WNV RNA.

In summary, we were successful in detecting WNV RNA in wastewater across multiple states, often before documented human infection or other ArboNET-reported surveillance methods. Future studies could determine the practicality of WNV WS to support WNV prevention and control in specific jurisdictions. Our results highlight the potential value of WS for WNV, particularly in locations with limited resources for surveillance. 
